# Automated telephone interventions for problematic alcohol use in clinical and population samples: a randomized controlled trial

**DOI:** 10.1186/s13104-017-2955-4

**Published:** 2017-11-28

**Authors:** Claes Andersson, Mikael Gajecki, Agneta Öjehagen, Anne H. Berman

**Affiliations:** 1 Department of Criminology, Faculty of Health and Society, Malmö University, 205 06 Malmö, Sweden; 2 Centre for Psychiatry Research, Department of Clinical Neuroscience, Karolinska Institutet, & Stockholm Health Care Services, Stockholm County Council, Norra Stationsgatan 69, 11364 Stockholm, Sweden; 3 Faculty of Medicine, Department of Clinical Sciences Lund, Psychiatry, Lund University, Lund, Sweden

**Keywords:** Alcohol, Hazardous, Dependence, Randomized, Intervention, Telephone, Automated, Outpatient, Psychiatry, Addiction, Help seekers

## Abstract

**Objective:**

The primary objective was to evaluate 6-month outcomes for brief and extensive automated telephony interventions targeting problematic alcohol use, in comparison to an assessment-only control group. The secondary objective was to compare levels of problematic alcohol use (hazardous, harmful or probable dependence), gender and age among study participants from clinical psychiatric and addiction outpatient settings and from population-based telephone helpline users and Internet help-seeker samples.

**Results:**

The Alcohol Use Disorders Identification Test (AUDIT) was used for screening of problematic alcohol use and 6-month follow-up assessment. A total of 248 of help-seekers with at least hazardous use (AUDIT scores of ≥ 6/≥ 8 for women/men) were recruited from clinical and general population settings. Minor recruitment group differences were identified with respect to AUDIT scores and age at baseline. One hundred and sixty persons (64.5%) did not complete the follow-up assessment. The attrition group had a higher proportion of probable dependence (71% vs. 56%; p = 0.025), and higher scores on the total AUDIT, and its subscales for alcohol consumption and alcohol problems. At follow up, within-group problem levels had declined across all three groups, but there were no significant between-group differences.

*Trial registration* ClinicalTrials.gov NCT01958359, Registered October 9, 2013. Retrospectively registered

## Introduction

Problematic alcohol use is prevalent in the general population, with higher prevalence in clinical settings such as psychiatry [1, 2]. Only about one in five individuals in the population seek treatment [3–5], a low proportion particularly due to stigma [6, 7]. Brief interventions to reduce problematic alcohol use have been shown to yield small but consistent behavior change effects across a variety of settings [8]. Over the past decade, digital brief interventions have been shown to reach individuals who otherwise might not seek treatment, and yield low, but positive treatment effects [9]. Qualitative research suggests that digital interventions might actually be preferred by individuals with problematic alcohol use [10, 11]. Specific research concerns in the literature on digital interventions for problematic alcohol use include high attrition rates [12, 13], and assessment reactivity, meaning that simply asking about alcohol use can lead to outcomes similar to intervention effects [14–16].

The current randomized trial evaluated brief intervention for problematic alcohol use via automated telephony, a digital system with high potential because of simplicity, accessibility and low costs [17, 18]. Automated telephony has primarily been used for screening and follow-up assessments [19–21], and in a number of feasibility studies [22–30]. Intervention studies have shown primarily positive results [31–33], but also negative results [34]. For this study, Swedish help-seekers from the general population and from clinical settings were recruited. The interventions used were modified versions of previously evaluated interventions [31, 35, 36].

The primary study objective was to evaluate 6-month outcomes for brief and extensive automated telephony interventions targeting problematic alcohol use, in comparison to an assessment-only control group. Secondary objectives were to compare levels of problematic alcohol use (hazardous, harmful or probable dependence), gender and age among study participants from clinical psychiatric and addiction outpatient settings and from population-based telephone helpline users and Internet help-seeker samples.

## Main text

### Methods

This was a randomized controlled trial with three parallel groups: (1) 1-month brief intervention; (2) 1-month extensive intervention; and (3) assessment only controls, with 6-month follow-up. An automated telephony system was pre-programmed to conduct all assessments and interventions.

The study was conducted in Sweden between February 2011 and January 2014. Participants were help-seekers from outpatient clinical settings and from the general population. Following a nationwide invitation letter, 14 psychiatric outpatient clinics agreed to participate in the study. Outpatients in addiction treatment were recruited from five clinics, two in the capital area and three in southern Sweden. All outpatients were recruited through waiting room advertisements. From the general population, individuals calling a national alcohol helpline during unmanned hours were offered brief information on the study if they selected this option from the telephone menu [37]. Individuals seeking information about help for alcohol-related problems over the Internet in Swedish were offered brief information about the study through Google ads restricted to Sweden.

All information was in Swedish, formulated to elicit interest among persons concerned about their alcohol use, and interested in participating in a research project. Participants from each group were referred to different designated telephone numbers for each group indicating recruitment setting. Complete study and registration information was available through each number.

Following informed consent, participants were asked to report gender and age. Both baseline and the 6-month follow-up assessments included an automated telephony version of the Swedish version of the Alcohol Use Disorders Identification Test, AUDIT [38], consisting of ten items, each scored with 0–4 points and yielding a maximum score of 40. The items cover two domains: alcohol consumption (AUDIT-C, items 1–3), and alcohol problems (items 4–10) [39]. Items were formulated to cover the standard time frame of 12 months at baseline, and reformulated at follow-up to cover the study period of 6 months. The cut-off used for hazardous drinking was ≥ 6 for women and ≥ 8 for men [38]. Harmful alcohol use was defined as a score between 16 and 19, and probable dependence was defined as ≥ 20 points for both genders [40].

Ability to understand spoken Swedish, age of ≥ 18 years, and AUDIT scores indicating at least hazardous use were inclusion criteria. Information to any excluded participants included a recommendation to contact the national alcohol helpline during opening hours. Included participants were automatically randomized and immediately informed about group allocation. Participants were blind to the intervention allocation but were aware that they could be assigned to a control group. Control group participants were only given information about the forthcoming 6-month follow-up.

The brief intervention began with feedback about the participant’s hazardous alcohol use. Participants were asked to set an individual goal for alcohol use: either abstinence or drinking below the levels recommended by national public health guidelines for risky daily and weekly consumption [41]. To keep track of drinking, automated follow-up calls were made each Monday during 4 weeks. Each follow-up included retrospective assessments of daily alcohol use during the preceding week, followed by feedback on the summarized number of drinks consumed over the past week.

The extensive intervention also provided feedback on the participant’s hazardous alcohol use, with information on the national public health guidelines for weekly and daily consumption. Participants were then asked to set an individual goal, either to reduce drinking or attain abstinence. Participants wanting to reduce drinking were directed to a menu of exercises including spoken texts on the advantages and disadvantages of drinking and vignettes presenting different strategies; e.g., keeping an alcohol calendar or attending an Alcoholics Anonymous meeting. Participants choosing abstinence were directed to a menu of exercises, such as learning to refuse alcohol in social situations, and relaxation/mindfulness exercises. Each call started and ended with self-ratings on the participant’s concern about alcohol consumption, with feedback at the end of the conversation on whether their concern had changed after completing the exercises. Participants were asked to practice the tasks and exercises suggested, and they had unlimited access to the platform during 4 weeks, with automated follow-up calls made each Monday.

The pre-trial power analysis based on repeated measures analysis of variance indicated that 22 participants were required in each group (brief intervention, extensive intervention and controls, from each of the psychiatry, addiction and population-based settings) assuming a small effect of d = 0.20, with a power of 0.90. Taking probable attrition into account, we estimated that double this number; i.e., 264 participants, would be needed to ensure a robust analysis. SPSS version 22 was used for all statistical calculations.

Descriptive statistics are presented as means and standard deviations (SD) for continuous variables and as counts for categorical data. Mann–Whitney non-parametric tests were used for continuous variables and Fisher’s exact test for categorical variables. The outcome variables were also analyzed with analysis of covariance, ANCOVA [42]. Each participant’s follow-up score was adjusted for the baseline score by analyzing the follow-up assessment as the dependent outcome, with the baseline score as a covariate, and the comparison groups regarded as fixed variables. The *b* coefficient is the estimated difference between two treatment groups, presented with a 95% confidence interval (CI). All tests were two-tailed. Test results with *p* values of ≤ 0.05 were regarded as statistically significant. Evaluators were blinded regarding participants’ randomization allocation.

### Results

**Fig. 1:**
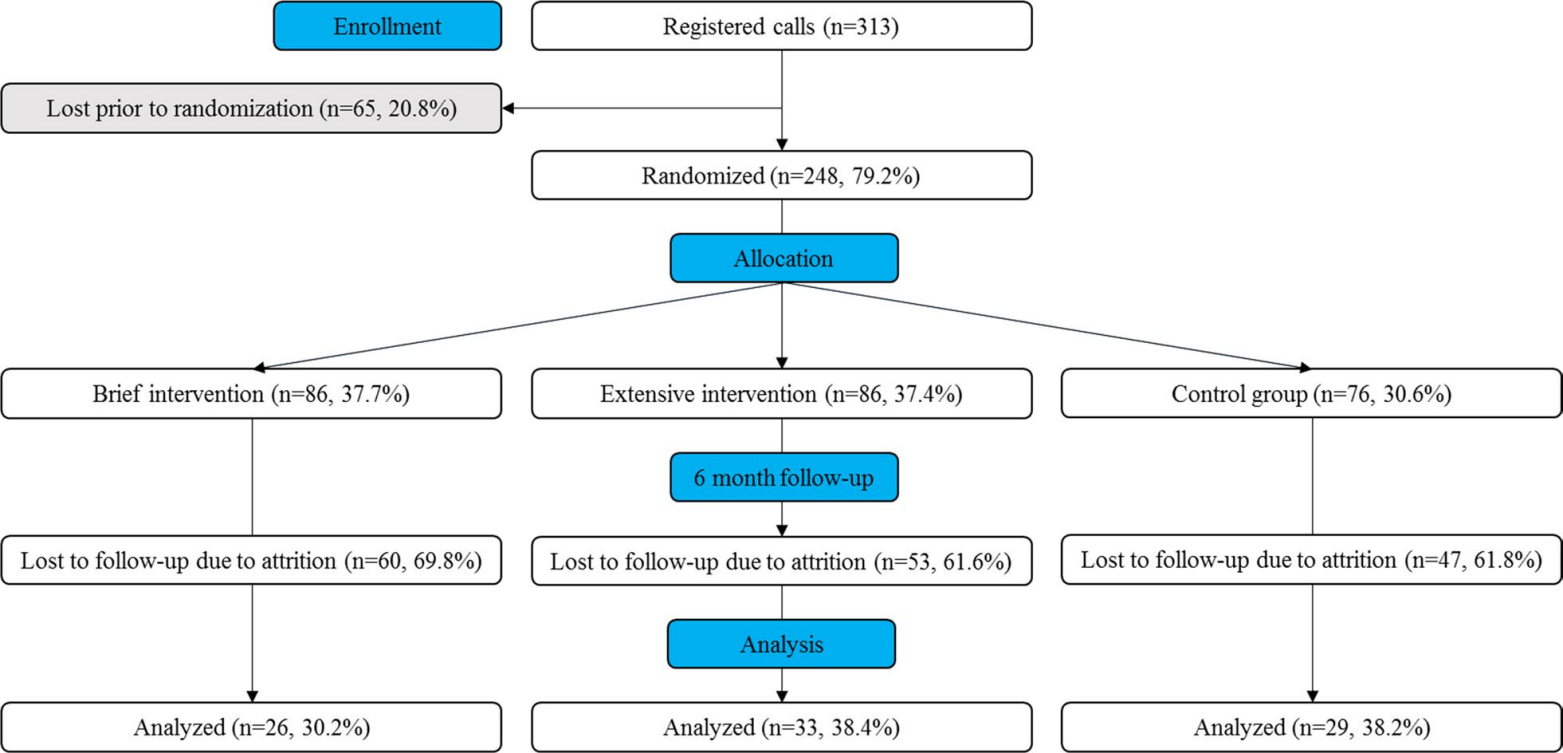
Consort diagram of the trial. Shaded areas were not included in analyses

A participant flow chart is shown in Fig. 1. The automated system registered calls from 313 individuals, with 65 lost prior to randomization and none excluded due to non-hazardous drinking. Available data showed no differences between individuals lost prior to randomization, and included participants. A total of 248 persons were randomized; see Table 1 for a comparison of baseline characteristics by recruitment setting.

**Table 1:** Baseline data by recruitment setting; patients in psychiatry and addiction outpatient treatment, telephone helpline and internet help-seekers

	Outpatients in clinical treatment			Help-seekers from the general population		
	Total (n = 56)	Psychiatry (n = 45)	Addiction (n = 11)	Total (n = 192)	Helpline (n = 3)	Internet (n = 189)
Intervention group/control group (%)	42/14 (75/25)	32/13 (71/29)	10/1 (91/9)	130/62 (68/32)	3/0 (100/0)	127/62 (67/33)
Brief/extensive intervention (%)	22/20 (52/48)	16/16 (50/50)	6/4 (60/40)	64/66 (49/51)	3/0 (100/0)	61/66 (48/52)
Men/women (%)	24/32 (43/57)	17/28 (38/62)	7/4 (64/36)	94/36 (49/51)	1/2 (33/66)	93/96 (49/51)
Age, mean (SD)	40.5 (14.0)	37.8 (13.1)^a, b^	51.6 (12.2)^a, c^	43.8 (13.3)	25.0 (6.6)^c, d^	44.1 (13.2)^b, d^
AUDIT						
Probable dependence (%)	37 (66)	28 (62)	9 (82)	125 (65)	2 (67)	123 (65)
Harmful use (%)	8 (14)	6 (13)	2 (18)	36 (19)	0 (0)	36 (19)
Hazardous use (%)	11 (20)	11 (25)	0 (0)	31 (16)	1 (33)	30 (16)
Total score, mean (SD)	22.2 (7.1)	21.4 (7.3)	25.5 (5.3)^e^	21.7 (5.5)	17.7 (9.5)	21.7 (5.5)^e^
AUDIT-C score, mean (SD)	8.7 (2.3)	8.6 (2.3)^f^	10.1 (1.8)^f, g^	8.8 (1.8)	7.7 (4.5)	8.9 (1.8)^g^
Alcohol problem scale, mean (SD)	13.4 (5.2)	12.9 (5.3)	15.4 (4.3)	12.8 (4.3)	10.0 (5.2)	12.8 (4.3)

p values: ^a^ 0.005; ^b^ 0.006; ^c^ 0.011; ^d^ 0.017; ^e^ 0.041; ^f^ 0.046. ^g^ 0.016

Of the 248 included participants, 160 (64.5%) did not complete the follow-up assessment. The attrition group had a higher proportion of probable dependence (71% vs. 56%; p = 0.025), a higher total AUDIT score (22.5 ± 6.0 vs. 20.6 ± 5.5; p = 0.014), a higher AUDIT-C score (9.0 ± 2.0 vs. 8.5 ± 1.9; p = 0.050) and a higher score on the alcohol problem scale (13.5 ± 4.6 vs. 12.1 ± 4.2, p = 0.019) compared to those remaining in the study (not tabulated). Sub-group analyses by gender, age and alcohol severity, as well as attrition/retention and recruitment setting groups, revealed that higher rates of probable dependence in the attrition group were associated with being older than the mean age of 43 years and being of male gender. Higher total AUDIT and alcohol problem subscale scores were associated with being older than the mean age, being of female gender, and membership in the Internet help-seeker group. Higher AUDIT-C scores were associated with being younger than the mean age.

Outcome analyses are shown in Table 2 for the 88 subjects who completed the follow-up assessment. Proportions of levels of problematic drinking at baseline and at follow-up were analyzed along with simple trajectory movements over time (no change, impaired, improved), showing that two-thirds of the participants improved; i.e., reduced their alcohol use to a lower category. No overall differences in outcome were identified by group allocation. However, analyses of AUDIT total scores and sub-score ratings in both intervention groups showed nominally higher figures, suggesting that greater improvement in comparison to the control group might occur in a larger sample; the extensive intervention group showed a similar trend towards greater improvement compared to the brief intervention group. Separate outcome analyses were conducted by gender, age, and recruitment setting, showing no differences.

**Table 2:** Outcome analyses of AUDIT scores: proportions of probable dependence; harmful use, hazardous use, non-risky use, trajectories (no change, impaired, improved), total and subscale ratings

	Intervention groups (n = 59)			Control group (n = 29)
	Total (n = 59)	Brief (n = 26)	Extensive (n = 33)	
AUDIT, probable dependence (%)				
Baseline	33 (56)	15 (58)	18 (55)	16 (55)
Follow-up	11 (19)	5 (19)	6 (18)	6 (21)
AUDIT, harmful use (%)				
Baseline	12 (20)	7 (27)	5 (15)	8 (28)
Follow-up	7 (12)	3 (12)	4 (12)	6 (21)
AUDIT, hazardous use (%)				
Baseline	14 (24)	4 (15)	10 (30)	5 (17)
Follow-up	26 (44)	14 (54)	12 (36)	11 (37)
AUDIT, non-risky use (%)				
Baseline	0 (0)	0 (0)	0 (0)	0 (0)
Follow-up	15 (25)	4 (15)	11 (34)	6 (21)
AUDIT, trajectories (%)				
No change	19 (32)	9 (35)	10 (30)	10 (34)
Impaired	1 (2)	1 (4)	0 (0)	2 (7)
Improved	39 (66)	16 (61)	23 (70)	17 (59)
AUDIT, total score, mean (SD)				
Baseline	20.8 (5.9)	21.3 (5.8)	20.3 (6.1)	20.2 (4.6)
Follow-up	12.5 (8.5)	14.1 (7.3)	11.2 (9.3)	13.7 (8.3)
Change score	− 8.3 (7.7)	− 7.2 (8.1)	− 9.1 (7.4)	− 6.5 (9.3)
ANCOVA, *b* (95% CI)				
Intervention vs. control	− 1.5 (− 5.1; 2.0)	− 1.6 (− 4.4; 4.1)	− 2.6 (− 6.8; 1.6)	
Extensive vs. brief			− 2.2 (− 6.2; 1.8)	
AUDIT-C score, mean (SD)				
Baseline	8.5 (2.0)	8.7 (1.9)	8.3 (2.0)	8.7 (1.8)
Follow-up	5.1 (3.3)	5.5 (2.8)	4.8 (3.6)	6.2 (3.5)
Change score	− 3.4 (3.4)	− 3.1 (3.0)	− 3.6 (3.6)	− 2.5 (3.5)
ANCOVA, *b* (95% CI)				
Intervention vs. control	− 1.0 (− 2.5; 0.5)	− 0.6 (− 2.3; 1.1)	− 1.3 (− 3.0; 0.5)	
Extensive vs. brief			− 0.6 (− 2.3; 1.0)	
Alcohol problem scale, mean (SD)				
Baseline	12.3 (4.5)	12.7 (4.4)	12.0 (4.7)	11.5 (3.6)
Follow-up	7.4 (5.7)	8.6 (5.3)	6.5 (5.9)	7.6 (5.3)
Change score	− 4.9 (5.1)	− 4.1 (5.8)	− 5.6 (4.4)	− 4.0 (6.0)
ANCOVA, *b* (95% CI)				
Intervention vs. control	− 0.6 (− 1.7; 2.9)	− 0.7 (− 3.6; 2.2)	− 1.4 (4.0; 1.2)	
Extensive vs. brief			− 1.7 (− 4.2; 0.9)	

No significant results were identified

### Discussion

Previous studies have shown small but consistent results in favor of brief interventions [8]. In the present study both intervention efficacy and intervention intensity were limited by insufficient sample size. No significant between-group differences were established. All study groups reduced problematic drinking over time, suggesting that the intensive brief interventions did not contribute to effects beyond assessment reactivity [14–16].

Baseline data showed high rates of probable dependence in both patients and the general population, though some differences were evident in relation to age and alcohol use. The present study confirms that individuals from different populations may be attracted by digital brief interventions [10, 11]. As in several previous studies on digital interventions, power in the present study was adversely affected by high dropout rates related to increased levels of problematic drinking [13].

One significant strength of this study is that it is one of few studies evaluating automated telephony by offering brief intervention targeting problematic alcohol use. Further, this study was unique in that it included both clinical and population samples, where both indicated concern about their drinking via study registration. It is worth noting that all prospective participants had at least hazardous alcohol use and that a high proportion showed probable dependence.

## Limitations

1. The most important shortcoming in this study is that it became clearly underpowered due to higher attrition than expected. Insufficient sample size meant that the primary research question could not be adequately evaluated. The low interest in completing the study was related to level of problematic alcohol use, suggesting that the intervention might have been less suitable for individuals with more severe problems. These individuals might need interventions with longer duration, possibly with counselor guidance, in order to benefit from digital interventions for problematic alcohol use [43].
2. Due to unexpected technical shortfalls, information was unavailable on participants’ opinions regarding automated telephony as a format for brief interventions, as well as opinions on the interventions themselves. Likewise, information was unavailable on the extent to which participants actually used the interventions, including exercises chosen for practice, and exercise follow-ups during the intervention period. This information could have been of clinical as well as scientific importance.
3. The present study relies on self-reported data. Though the AUDIT is a well-established instrument, clinical interview-based diagnosis of alcohol use disorders, preferably complemented with biomarker data, would have been more reliable.
4. We were unable to estimate the total number of patients at participating clinics, the total number of help seekers contacting the national helpline, as well as the sample accessing information about the study via the Internet. These shortcomings meant that data from the present study could not be used to estimate the representativity of problematic alcohol use in the four groups.
5. Another aspect limiting our capacity to estimate potential participant interest in the study is that data were missing on the number of incoming calls for registration.

## Abbreviations

AUDIT: Alcohol Use Disorders Identification Test
SD: standard deviation
ANCOVA: analysis of covariance
B: estimated difference between two treatment groups
CI: confidence interval
p: probability value
